# Digitally Mediated Occupational Therapy to Increase Physical Activity in Urban and Rural Breast Cancer Survivors: Protocol for a Single-Arm Feasibility Trial

**DOI:** 10.2196/73554

**Published:** 2025-09-26

**Authors:** Tara C Klinedinst, Zachary C Pope, Michael C Robertson, Nadia Stanley, Audrey Wint, Christina Henson, Darla E Kendzor

**Affiliations:** 1 Department of Rehabilitation Sciences College of Allied Health The University of Oklahoma Health Campus - Tulsa Tulsa, OK United States; 2 Department of Internal Medicine OU-TU School of Community Medicine Tulsa, OK United States; 3 TSET Health Promotion Research Center Stephenson Cancer Center The University of Oklahoma Health Campus Oklahoma City, OK United States; 4 Department of Health Promotion Sciences Hudson College of Public Health The University of Oklahoma Health Campus Oklahoma City United States; 5 Department of Family and Preventive Medicine College of Medicine The University of Oklahoma Health Campus Oklahoma City, OK United States; 6 Department of Radiation Oncology College of Medicine The University of Oklahoma Health Campus Oklahoma City United States

**Keywords:** Self-Determination Theory, muscle-strengthening exercise, telehealth, supportive care, mobile phone

## Abstract

**Background:**

The 5-year survival rate for breast cancer (BC) has increased in recent years. However, functional limitations associated with BC treatment (eg, loss of strength, fatigue, and lymphedema) often have far-reaching effects on survivors’ physical and mental health. Aerobic physical activity (PA) and muscle-strengthening exercise (MSE) can reduce functional limitations, and occupational therapy (OT) can support these health-promoting behaviors after treatment. Yet, barriers to access among BC survivors (eg, time burden and distance to the OT clinic) limit participation in OT programing. This is particularly true in Oklahoma, where 33% of residents live in rural counties. Digital technologies (eg, telehealth) can help urban and rural BC survivors circumvent these barriers.

**Objective:**

We are investigating the feasibility of a novel OT program among urban and rural BC survivors that features (1) 8 once-weekly telehealth OT sessions targeting constructs grounded in Self-Determination Theory (SDT), and (2) self-regulatory strategies known to support aerobic PA and MSE in BC survivors including self-monitoring via a wearable PA tracker, goal setting, and the provision of timely feedback.

**Methods:**

This is a single-arm feasibility trial. We are recruiting 38 BC survivors using community-based recruitment approaches and via referral from collaborating oncologists. Participants include individuals who have undergone primary treatment and/or breast-conserving surgery or mastectomy for BC in the last 24 months and who do not meet recommended PA levels at the time of enrollment. We will assess self-reported program acceptability and feasibility via recruitment rates, study retention, and protocol adherence. We will also evaluate program safety by tracking BC-related lymphedema events, musculoskeletal injuries, and other adverse events. Finally, we will assess changes in aerobic PA, MSE, and health-related quality of life during the program period using accelerometry and self-report measurement tools.

**Results:**

We received funding in March 2024 and institutional review board approval in September 2024. We began recruiting in November 2024. We anticipate completing data collection in early 2026. We hypothesize that the SDT-grounded OT program will be acceptable, feasible, and safe. We also expect pre- to post-program improvements in (1) SDT-informed determinants of PA, (2) levels of aerobic PA and MSE engagement, and (3) health-related quality of life.

**Conclusions:**

The novel OT program under investigation is designed to decrease barriers to engaging in aerobic PA and MSE among people who have undergone various BC treatments. It is centered on facilitating a successful transition from active treatment to the posttreatment period and combines OT with the benefits of telehealth delivery and health behavior change theory. This program is amenable to wide-scale dissemination and, if shown to be acceptable and feasible, will represent a promising approach to supportive cancer care.

**Trial Registration:**

ClinicalTrials.gov NCT06671730; https://clinicaltrials.gov/study/NCT06671730

**International Registered Report Identifier (IRRID):**

DERR1-10.2196/73554

## Introduction

### Background

The 5-year breast cancer survival rate across all races is 90% [1]. However, given that most breast cancer diagnoses lead to either breast-conserving surgery or mastectomy, survivors tend to experience considerable functional limitations caused by both cancer and its treatment. These functional limitations include lymphedema, reduced range of motion, fatigue, and pain, and often impact survivors’ physical and mental health-related quality of life (HRQoL) [2]. Unfortunately, these functional limitations can restrict participation in health-promoting activities, such as aerobic physical activity (PA) and muscle-strengthening exercise (MSE) [3], placing survivors at an increased risk of morbidity and premature mortality [3,4]. Therefore, developing programs that reduce functional limitations and engage breast cancer survivors in aerobic PA and MSE is critical to improving health and well-being while decreasing risk factors.

PA is safe and beneficial for most cancer survivors [2,5] and can improve HRQoL via increased self-efficacy and physical, emotional, social, and functional well-being [6]. Yet, less than 20% of breast cancer survivors meet recommended aerobic PA and MSE guidelines [7-9]. Qualitative research highlights that breast cancer survivors are uncertain about how to integrate aerobic PA and MSE into their lives after transitioning out of formal care settings. Support is needed to help this population with lingering functional limitations to transition into sustained, self-directed PA and MSE in community- and home-based settings [10].

**Figure 1:**
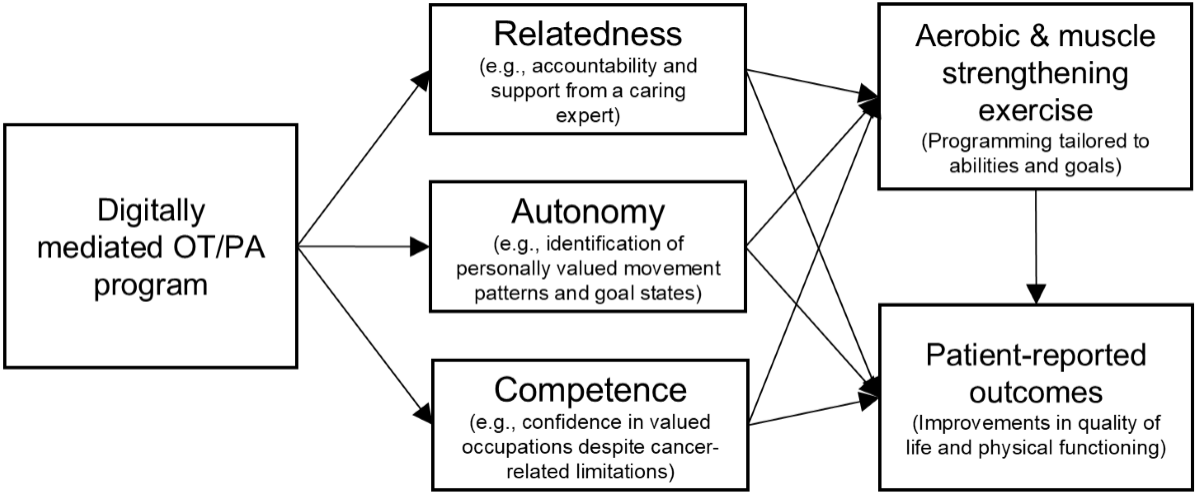
Conceptual model for a Self-Determination Theory–informed occupational therapy program for sustained physical activity. OT: occupational therapy; PA: physical activity.

Occupational therapy (OT) is uniquely capable of addressing the functional limitations that restrict PA and MSE in breast cancer survivors through a combination of rehabilitation for physical function, adaptation of activity for functional limitations, environmental modification, and through developing supportive habits and routines. The goal of OT for cancer survivors is to safely and independently return to activities of daily living following cancer treatment. As such, OT is a critical posttreatment component for breast cancer survivors, who are likely experiencing multiple functional limitations. Self-Determination Theory (SDT) posits that the satisfaction of people’s core psychological needs (ie, autonomy, competence, and relatedness) facilitates the formation of autonomous motivations for health-related behaviors (ie, enjoyment, interest, identity, and values) [11,12]. Importantly, SDT has been proposed as an ideal theoretical framework for guiding OT (Figure 1) [13,14]. Grounding OT treatment in SDT can support sustained, self-directed PA, and MSE through orienting survivors toward growth and development.

Digitally mediated OT is uniquely suited to helping breast cancer survivors (in particular, rural survivors) transition to sustained community- and home-based PA after undergoing primary breast cancer treatments (eg, lumpectomy and partial or full mastectomy), dramatically increasing the accessibility of expert-provided PA and MSE programing. The use of digital technologies, such as telehealth, can help urban and rural breast cancer survivors circumvent challenges (eg, time burden and distance to OT clinic) to receiving OT programing [15-17]. In Oklahoma, breast cancer continues to pose a significant health challenge, with incidence rates around 124.5 per 100,000 women [18]. Survivors in rural areas often face barriers to care, including long travel distances, limited access to specialists, and socioeconomic constraints. To address these challenges, telerehabilitation has emerged as a promising solution. Emerging evidence suggests that technology-mediated OT is feasible for urban and rural breast cancer survivors [19,20]; however, a review of the literature highlights a lack of accessible and individualized programs to improve PA engagement in this population [21].

To that end, we are conducting an Obesity-Related Behavioral Intervention Trials Phase II trial [22] to investigate the acceptability and feasibility of a novel 8-week OT program among 38 urban and rural breast cancer survivors that features (1) 8 once-weekly telehealth OT sessions, and (2) self-regulatory strategies known to support aerobic PA and MSE adoption and maintenance [23,24], emphasizing components of the SDT, and the provision of a wearable device (eg, a fitness tracker) programed to supplement individually tailored goals. Our specific aims, hypotheses, and milestones are detailed in the following sections.

### Aim 1

Assess participants’ perceptions of the acceptability and usefulness of the SDT-grounded, OT-based aerobic PA and MSE program (hereafter “program”). Hypothesis 1: participants will report the program’s components to be acceptable, useable, and useful. Milestone 1a: thematic analyses of qualitative data will suggest that the program was enjoyable and improved physical and mental well-being. Milestone 1b: quantitative assessments of items reflecting program acceptability will have a median of 5 or higher on a 7-point Likert-type scale.

### Aim 2

Evaluate program feasibility and safety. Hypothesis 2: the program will be feasible and safe as demonstrated by satisfactory participant retention and adherence to program components and no serious adverse events related to the program. Milestone 2a: at least 70% of participants who complete baseline measures will subsequently complete post-program measures. Milestone 2b: participants will average ≥80% attendance at OT sessions. Milestone 2c: the frequency of breast cancer–related lymphedema, musculoskeletal injuries, and any adverse events will be at or below normative values—indicative of a safe program [25-27].

### Exploratory Aims

Evaluate pre- to post-program changes in (1) SDT-related constructs (autonomy, competence, and relatedness) as key mechanisms of action, (2) aerobic PA and MSE, and (3) patient-reported outcomes (PROs, eg, physical function, anxiety, and pain intensity and interference) and goal attainment. Exploratory hypotheses: pre- to post-program changes in SDT-related components, aerobic PA and MSE behaviors, and PROs and goal attainment will be favorable. Milestone 3a: measures of perceived autonomy, competence, and relatedness—key SDT components in the context of aerobic PA and MSE—will improve from pre- to post-program [28]. Milestone 3b: participants will demonstrate a median aerobic PA increase of ≥10 minutes per day and will engage in at least 2 MSE bouts per week by post-program. Milestone 3c: an average pre- to post-program change of 2-6 t-score points, dependent on the PRO being assessed, as measured by the Patient-Reported Outcomes Measurement Information System (PROMIS), with most participants (≥75%) achieving their pre-program goals by post-program.

## Methods

### Participants and Recruitment

Participants (N=38) in this feasibility trial are urban and rural breast cancer survivors within Oklahoma City and the greater state of Oklahoma. We define rurality using the US Department of Agriculture Economic Research Service’s 2020 Rural-Urban Commuting Area codes [ie, metropolitan (1-3) vs micropolitan, small town, and rural (4-10)]. We are conducting rolling recruitment, with a target accrual rate of approximately 5 participants per month. We are recruiting English-speaking participants from local survivorship organizations and events, by approaching patients and posting flyers at the Stephenson Cancer Center, through The University of Oklahoma Health Sciences (OUHS) social media advertising, and via direct referral from a collaborating oncologist. We are evaluating the eligibility of prospective breast cancer survivor participants using a screening questionnaire administered via REDCap (Research Electronic Data Capture). Qualifying breast cancer survivors are then contacted by a study team member to set up their baseline consenting and orientation appointment. Eligible participants must be aged 18 years and older, able to speak and read English and provide informed consent, must have received a histologically confirmed diagnosis of invasive breast carcinoma, have undergone primary treatment and/or surgery for breast cancer in the last 24 months, own a smartphone or computer with internet access, and be willing to participate in once-weekly telehealth-delivered OT sessions for 8 weeks. Individuals are ineligible if they are currently undergoing chemotherapy or radiation as primary cancer treatment, planning or preparing for surgery as primary treatment or as a reconstruction procedure in the next 3 months, have a distant metastasis, or report a Physical Activity Readiness Questionnaire score that indicates that PA may potentially be unsafe, unless the participant produces a signed doctor’s note. Additionally, individuals are eligible if they are already engaging in ≥75 minutes per week of vigorous-intensity PA; ≥150 minutes per week of moderate-intensity PA, or an equivalent combination of both over the last 3 months; if they are currently seeking OT or physical therapy treatment; or if they are a prisoner, pregnant, or planning to become pregnant. Although a power analysis was not appropriate due to our study’s pilot classification, a sample size of 38 participants was chosen, given that, anticipating attrition, this sample size (1) facilitates the detection of feasibility issues and provides sufficient data for useful parameter estimates, and (2) strikes a practical balance between our scientific objectives and available resources [29]. This trial is registered on ClinicalTrials.gov (NCT06671730).

### Program Description

**Figure 2:**
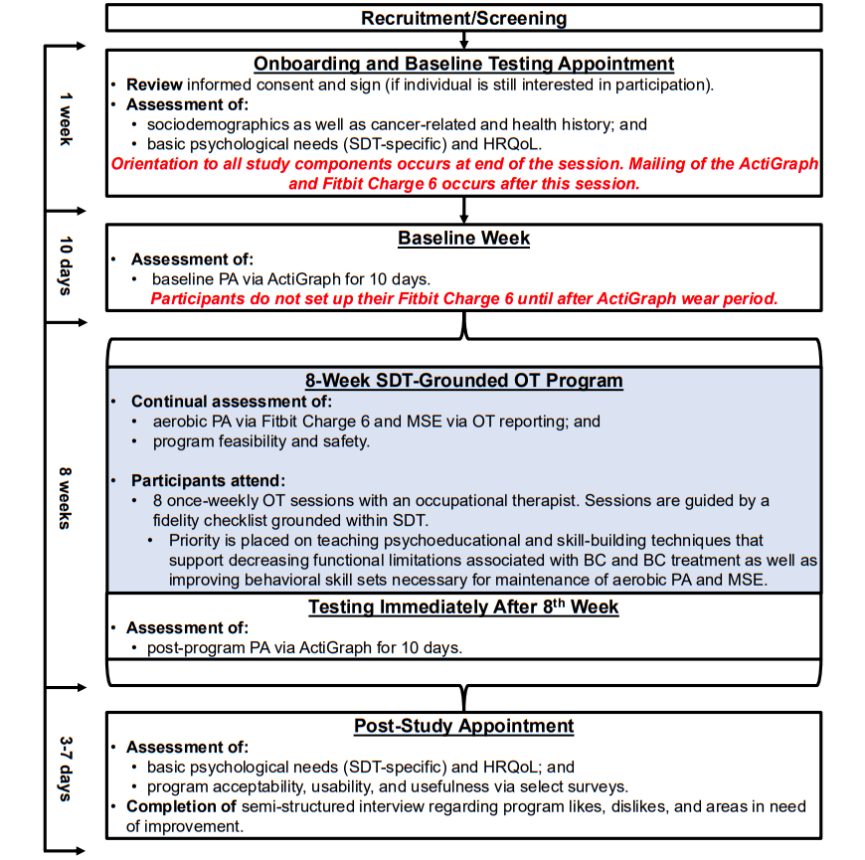
Participant timeline. BC: breast cancer; HRQoL: health-related quality of life; MSE: muscle-strengthening exercise; OT: occupational therapy; PA: physical activity; SDT: Self-Determination Theory.

The telehealth-based OT program is grounded in SDT (Figure 1), with the participant timeline throughout the study presented in Figure 2. The program is designed to help breast cancer survivors transition from recovery after surgery to achieving levels of aerobic PA and MSE that are recommended according to each participant’s specific treatment course and desired outcomes [2,10,30]. Participants are engaging in 8 once-weekly OT sessions with a licensed occupational therapist using videoconference software (Zoom; Zoom Communications). Each session lasts up to about an hour and is centered on psychoeducational and skill-building techniques to (1) reduce functional limitations associated with cancer and its treatment and (2) develop behavioral skills for maintenance of aerobic PA and MSE. We chose an 8-week program duration with once-weekly sessions because this duration and cadence aligns with typical OT treatment plans and was deemed to provide sufficient time to support meaningful changes in physical and psychological health outcomes while maintaining participant engagement, minimizing attrition, and ultimately supporting program scalability.

During the first session, participants work with the occupational therapist to choose exercise equipment from a list of options (Multimedia Appendix 1) that supports their preferences and valued occupations (value of up to US $100). The selected equipment is then mailed to the participant by the research team. During the second OT session, the occupational therapist works with participants to identify and set goals related to aerobic PA and MSE choices that align with their values, interests, and strengths. Participants then use their selected exercise equipment to engage in tailored aerobic PA and MSE throughout the remainder of the sessions, with the occupational therapist also tracking participant attendance at each week’s OT session for each participant as one of several adherence measurements.

Participants are also provided with a Fitbit Charge 6 fitness tracker. The occupational therapist has access to these trackers’ data via a cloud-based platform, Fitabase (Fitabase; Small Steps Lab LLC), that queries the Fitbit application programing interface. During the weekly OT sessions, the occupational therapist promotes self-regulatory techniques known to be useful for PA initiation and maintenance [12]. These include the provision of timely feedback on PA levels; encouraging specific, attainable goal setting (eg, planning gradual increases in step counts); use of strengths-based problem-solving; and action planning (eg, when and how participants plan to meet aerobic PA or MSE goals). Table 1 outlines the general tasks and emphases of each week’s OT session. Fidelity of each OT session is being evaluated using an established fidelity checklist method with occupational therapist and supervisor assessment [31]. Briefly, Zoom sessions are being videorecorded, and 20% of the sessions will be randomly selected for review and rating by a PhD-level occupational therapist (TCK) [31]. Furthermore, participants’ goal attainment throughout the program is being evaluated with the Goal Attainment Scale [32] embedded within the fidelity checklist. Finally, participants wear the ActiGraph GT3X accelerometer for 10 days before and after receiving the 8-week OT program. Wear instructions and a tracking log to document accelerometer wear patterns are being provided.

Following the 8-week program and completion of all other study tasks (eg, ActiGraph wear and poststudy survey), the research coordinator is conducting an end-of-study semistructured interview with each participant to better understand their program experience. The goal of this interview is to gauge the usability, usefulness, and enjoyability of the program, by asking questions such as “How useful was experiencing this OT and physical activity program for you?” and, “How could we improve upon the program?”

**Table 1:** Occupational therapy elements targeting self-determination theory constructs for sustained physical activity.

SDT^a^ construct			OT^b^ objectives and techniques
**Session 1**			
	Relatedness	A discussion regarding the participants’ overarching “why” for participating in the program is designed to facilitate mutual understanding between the therapist and the participant and support therapeutic rapport.	
	Autonomy	The participant is noted as the expert in their needs and desires and encouraged to take a lead role in their values-based goal setting while receiving support from the occupational therapist. Participants are encouraged to take a lead role in the selection of their study-provided exercise equipment.	
	Competence	Participants rate their confidence in their ability to achieve their values-based goals and discuss what they feel performing much better, somewhat better, somewhat worse, and much worse would look like with the occupational therapist. The occupational therapist suggests specific action planning processes to put in place to support values-based goal attainment.	
**Sessions 2-7**			
	Relatedness	The occupational therapist engages in a semistructured “grounding” process before each session that includes a focus on adopting the client’s perspective, using neutral language, building therapeutic rapport, and understanding the participant. The occupational therapist presents an evolving, thoughtfully curated OT program designed to help the participants meet their goals and support their values.	
	Autonomy	All sessions explicitly include evocation and discussion of participants’ overarching “why” for participating in the program. All sessions include a weekly goal-setting discussion centered on encouraging the participant to identify short-term goals that align with their valued occupations.	
	Competence	The evolving, personally tailored program is explicitly designed to “meet participants where they are” and support incremental improvement in valued movement patterns. Qualitative and quantitative information (eg, goal attainment scaling) helps the occupational therapist to modify programing to best meet participant’s changing needs, abilities, and interests. In each session, the occupational therapist refers back to the participant’s explanation of what they felt performing much better, somewhat better, somewhat worse, and much worse than last week’s goal would look like. This helps participants to recognize progress and select appropriate weekly goals. Participants then rate their confidence in their ability to achieve the current week’s values-based goals and discuss what performing much better, somewhat better, somewhat worse, and much worse would look like. The occupational therapist suggests specific action planning processes to put in place to support values-based goal attainment.	
**Session 8**			
	Relatedness	Session 8 features a semistructured maintenance and continuity plan discussion. In addition to many of the objectives and techniques used in sessions 2-7, the maintenance and continuity plan includes a discussion of positive changes observed by the occupational therapist and experienced by the participant over the course of the program.	
	Autonomy	In addition to many of the objectives and techniques used in sessions 2-7, the maintenance and continuity plan includes an evocation and discussion of participants’ overarching “why” for participating in the program and a discussion of “meaningful takeaways” experienced by the participants over the course of the program.	
	Competence	In addition to many of the objective and techniques used in sessions 2-7, the maintenance and continuity plan includes a discussion of how participants can maintain their momentum upon conclusion of the program.	

^a^SDT: Self-Determination Theory.

^b^OT: occupational therapy.

### Protocol Changes

We now have institutional review board (IRB) approval to recruit breast cancer survivors having completed any type of primary treatment for breast cancer within the last 24 months. Our previous criterion considered eligible only those who had undergone some type of breast-conserving surgery or a partial or full mastectomy within the last 12 months. Our discussions with participating breast cancer survivors as well as breast cancer survivors at local survivorship events informed this decision, with many noting marked limitations even in the absence of more aggressive treatment (eg, a full mastectomy).

### Outcomes, Measurements, and Analyses by Study Aim

We are collecting sociodemographic and medical information at baseline to describe our sample, including age, sex, race and ethnicity, education, height and weight, marital status, annual household income, date of breast cancer diagnosis and stage at diagnosis, type of treatments received, comorbidities (eg, type 2 diabetes), readiness to change, and overall health status.

#### Aim 1: OT Program Acceptability and Usefulness

To assess participants’ perceptions of the OT program’s acceptability, we are using qualitative and quantitative methodologies. For qualitative aim 1, a research team member is conducting semistructured interviews with each participant following study completion. Interviews are guided by a predetermined list of questions about the program meant to discern what program components participants liked (ie, found acceptable and useful) and disliked, as well as which aspects of the program they believe could be improved (ie, to enhance the program in future iterations). The research team member probes participants for further context regarding their answers as needed. Qualitative interviews are recorded and will be transcribed verbatim by a Health Insurance Portability and Accountability Act–compliant third party. Transcripts will be analyzed via thematic content analysis using qualitative analysis software [33]. The investigators will perform coding together for 2 transcripts to establish a consensus for coding procedures and then code the remaining transcripts independently. The thematic content analysis will include both deductive codes (ie, codes determined a priori that reflect program acceptability) and inductive codes (ie, codes reflecting emergent phenomenon noted by data analysts). Identified themes will then be discussed among the investigators, with discrepancies noted and resolved and final themes reported. Our quantitative aim 1 assessment of program acceptability and usefulness will be measured post-program. Participants will complete a questionnaire designed to provide insight into various facets of program acceptability (eg, perceived usability, usefulness, enjoyability, etc).

#### Aim 2: OT Program Feasibility and Safety

We are assessing program feasibility via measurements of recruitment rates, study retention, and protocol adherence. Recruitment rates are being reported as the proportion of breast cancer survivors enrolled in the study divided by the number of breast cancer survivors who complete the screening survey. Enrollment will be considered feasible if ≥70% of breast cancer survivors expressing interest, and who are eligible, ultimately enroll in the study. Study retention is being evaluated similarly, with the number of breast cancer survivors completing the program divided by the number that started. Retention will be considered feasible if ≥70% of breast cancer survivors who complete the baseline measures also complete measures post-program. The number of weekly telehealth-delivered OT sessions (out of 8 total sessions) that each participant attends is being used as the program adherence metric. Adherence will be considered feasible if at least 80% of participants average ≥80% attendance at their OT sessions. Assessments of program safety are being assessed by tracking any breast cancer–related lymphedema events, musculoskeletal injuries, and adverse events. The occupational therapist is tracking this information each week during each participant’s telehealth-delivered OT session. The program will be considered safe if these events occur at or below published normative values [25-27]. Results for program feasibility and safety will be reported descriptively as frequencies and percentages.

#### Exploratory Aims: SDT-Related Constructs, Aerobic PA and MSE, and PRO Changes

The proposed project is a pilot trial focused on testing a new interventional model and using observations to refine various program components. The project is not powered to detect statistically significant changes in outcomes. As such, while we propose measuring the outcomes below to properly inform future larger studies, we will calculate only descriptive statistics (ie, means and 95% CIs) for study outcomes [34,35]. Notably, we will use the Clopper-Pearson exact method to calculate all 95% CIs.

We are assessing participants’ pre- to post-program changes in SDT constructs through examinations of basic psychological needs satisfaction in exercise. We are operationalizing these constructs using the Basic Psychological Needs in Exercise Scale (BPNES) [36-39] The BPNES is an 11-item Likert-type scale that features 3 subscales that measure perceptions of autonomy, competence, and relatedness (1: “I don’t agree at all”; 5: “I completely agree”). Psychometric analyses have provided support for this scale’s reliability, discriminant validity, and measurement invariance across gender and various cultures, and we will score subscales individually using the recommended protocol [36-39]. We are also evaluating theory intervention fidelity at post-program using the Theory-Intervention Fidelity Test. This measure will allow us to assess participants’ perceptions of having experienced the SDT’s underlying mechanisms of behavior change (autonomy, competence, and relatedness) as well as meaning associated with the program’s aim (ie, to increase engagement in personally meaningful PAs). Responses are on a 5-point Likert-type scale (1: “Strongly disagree”; 5: “Strongly agree”). For the BPNES, we will calculate participant’s pre- to post-program change in perceived autonomy, competence, and relatedness (each construct will be calculated separately). Using these data, we will calculate participants’ mean change scores and corresponding 95% CIs. For the Theory-Intervention Fidelity Test, we will present data descriptively overall and for each construct (ie, autonomy, competence, relatedness, and meaning).

Measurements of pre- to post-program changes in aerobic PA are being measured using ActiGraph GT3X accelerometers. Daily durations of moderate-vigorous physical activity, light physical activity, and sedentary behavior (SB) will be reported. ActiGraph GT3X accelerometers have demonstrated evidence of validity for the measurement of free-living PA and SB [40]. To ensure the collection of reliable data, participants are wearing the accelerometers at baseline before the first OT session and donning of the Fitbit as well as during the 9th week following the OT program. Established protocols for placement and duration are being followed [41]. Wear instructions and an accelerometer wear log are also provided to all participants. We will exclude days not meeting established thresholds (≥10 hours per day of wear time, at least 3 weekdays, and 1 weekend day). We will then use established cut points to analyze PA durations per day for all valid days at each time point for each participant [42]. Daily durations of SB, light physical activity, and moderate-vigorous physical activity will be reported for baseline and the 9th week, with mean pre- to post-program changes and associated 95% CIs calculated. Notably, we may also use data from participant’s Fitbit Charge 6 to calculate more granular data on the above PA metrics as well as daily steps. Critically, Fitbits, and the Fitbit Charge Series, have good accuracy versus criterion for PA tracking in laboratory settings, with only slight underestimations in free-living settings (approximately 1 step per minute with outliers removed) [43,44]. Pre- to post-program changes in weekly MSE bouts are being tracked as the occupational therapist discusses PA goals with each participant during their once-weekly OT session. The occupational therapist’s notes will be used to determine the percentage of program weeks that breast cancer survivors meet the recommended 2 bouts of MSE per week [45].

Finally, we are evaluating pre- to post-program changes in HRQoL using PROs. We are assessing the following 8 components of physical and psychological health at baseline and post-program using the PROMIS: physical functioning, fatigue, anxiety, depression, pain interference, pain intensity, sleep impairment, and sleep disturbance [46]. Measurements on the PROMIS are on a 5-point Likert-type scale, with 5 reflecting worse outcomes on all components aside from physical function wherein a higher score reflects better physical function. These PROMIS measures have been observed as reliable tools for the assessment of these outcomes [47]. Baseline and post-program scores for each of the 8 PROMIS components will be calculated using established procedures and then converted to t-scores. A pre- to post-program change of 2-6 t-score points is considered a minimal important change relative to published normative values [47,48]. These PROMIS measures have been observed as reliable tools for the assessment of these outcomes [47]. We are measuring goal attainment via goal attainment scaling. Goal attainment scaling allows for reporting the extent to which a participant meets weekly goals using a 5-point scale (–2: performance was much worse than expected; +2: performance was much better than expected). Measurement of pre-post changes will be obtained through transforming responses into a single aggregate score using established protocols [32].

#### Missing Data

We are working to enhance participant retention and, therefore, data completeness through follow-up phone calls, incentives, and focusing on meaningful participant goals. Our research coordinator is also frequently checking for missing data, which will be flagged in our study database, located in REDCap. We are identifying and coding any missing data (ie, nonresponse to questions and missed study visits), looking for patterns, and identifying the appropriate assumptions of missing data (ie, missing completely at random, missing at random, or missing not at random). Due to the nature of the study, we will not need to perform sensitivity analyses; however, if we have multiple participants with incomplete data, we will decide between a complete case analysis and a last observation carried forward analysis at the recommendation of our biostatistician.

### Ethical Considerations

This study was approved by the OUHS IRB (number 16601). Participants are consented to the study prior to engaging in any study activity. Furthermore, participants can opt out of participation at any point during the study period. All participant data are deidentified, with data accessible to only those on the study team. Participants will be compensated with a US $50 gift card upon completion of all study tasks, US $100 worth of exercise equipment selected by the participant, and a Fitbit Charge 6.

### Privacy and Confidentiality

All data are being securely stored in REDCap. As this is a multiple principal investigator (MPI) study, the MPIs are jointly responsible for monitoring the safety environment of participants and ensuring that appropriate medical care and coverage are provided to all participants if necessary. The primary mentor (DEK) has also pledged to take responsibility to help lead and oversee all aspects of the research with human participants. The safety monitor (study oncologist; CH) has also pledged to oversee participant safety.

The MPIs are also responsible for monitoring procedures during conduct of the study for each participant, including eligibility, enrollment, data collection, evaluation of study outcomes, problems with informed consent, and participant safety and well-being. This is being undertaken under the guidance of the primary mentor. The plan includes (1) weekly review of screening results by the MPIs; (2) immediate reporting of adverse events by team members to the MPIs and then to the IRB, if needed; (3) quarterly review of collected data by the MPIs; (4) annual review by the MPIs, primary mentor, and OUHS IRB; and (5) to ensure safety, the study oncologist is providing an ad hoc review of adverse events. They will be sent this information within 2 weeks, or immediately for potentially serious or severe events.

Screening data are being reviewed weekly during recruitment periods by the MPIs. The MPIs are ensuring that any values indicating ineligibility or unsafe practices are flagged. Flagged participants may be excused from the study or instructed on safer practices, as needed. These decisions are being made in consultation with the study oncologist. We will not convene an external data safety and monitoring board. Should the study sponsor or the collaborating cancer center request that we do so, we will take advantage of protocols used in the past by investigators associated with our university.

Anticipated nonserious adverse events include nonserious injuries (eg, joint pain) and illnesses, minor discomfort, and possible loss of confidentiality. If an anticipated adverse event occurs, the MPIs and primary mentor will determine whether the event requires reporting to the IRB and to the study sponsor. The attribution and impact of each event on the overall project’s risk:benefit ratio will then be determined in consultation with the study mentor and oncologist.

All unanticipated, serious, fatal, and life-threatening adverse events will be immediately reported to the IRB within 24 hours of occurrence. The IRB, MPIs, and primary mentor are responsible for determining whether modifications are needed to the consent form or protocol based on the event. All protocol modifications will be reported to the IRB. If a determination is made that participants are exposed to unacceptably high risk in comparison with benefit, the study will be suspended until proper modifications are made for participant safety. Should cessation of study activities be required (temporarily or permanently), the program officer will be notified immediately.

## Results

This research study was funded in March 2024. We received approval from the OUHS IRB in September 2024, and we began recruiting in November 2024. We anticipate having all data collected by Spring 2026 and that the primary results manuscript submitted for publication in Summer 2026.

## Discussion

### Anticipated Findings

We anticipate that study participants will view the program favorably, and that the program will be safe and feasible to implement. We also anticipate that pre- to poststudy changes will demonstrate plausibility of the program’s effectiveness. Moreover, we expect that the program will compare favorably with previous and contemporary research concerning enjoyability, usefulness, and effects on determinants of sustained PA (eg, SDT constructs). This is because OT is oriented toward building capacity in participant-defined valued occupations; by integrating the promotion of active living in this autonomy-supportive context, we expect that this program will resonate with participants in ways that may support lasting behavior change.

### Pitfalls

In similar interventions, the most common pitfalls are adherence to the intervention and study processes, technology use, and safety of the participants. We will use the following strategies to improve adherence: (1) follow-up phone calls, (2) compensation via stipends and personalized exercise equipment, (3) virtual delivery of the program, and (4) the use of a participant goals–focused program that establishes therapeutic rapport. To address technological issues, we provide written guides for using the Fitbit and its app and technological assistance for Zoom. We also have a research coordinator available to troubleshoot any technological issues as they arise. We will ensure participant safety through standardized processes for (1) evaluating readiness for exercise, (2) reporting of adverse events, and (3) close monitoring by a licensed occupational therapist, a professional trained in safely supporting PA.

### Future Directions

We will use the observations from this feasibility trial to further refine this intervention protocol and scale the program for an effectiveness-implementation type 1 hybrid trial. This research aligns with the National Cancer Institute’s and American Cancer Society’s priority funding areas: using technology to improve access to survivorship care and testing evidence-based interventions that lead to reaching adequate levels of PA.

### Dissemination Plan

We will present our preliminary and final results, as well as exploratory observations, at major scientific meetings held by relevant scientific organizations. Furthermore, we will share information regarding our study through peer-reviewed publications that outline the study design and methods as well as overall study results. We will target top-tier journals for publishing and disseminating these observations. Importantly, we intend to work with the personnel at the Stephenson Cancer Center as well as the communication teams within the TSET Health Promotion Research Center and the OUHS to package our observations in research briefs for broad dissemination through all media avenues locally and globally, including social media.

### Strengths and Limitations

This study has a few notable strengths. First, our program addresses a critical transitional period for breast cancer survivors (acute posttreatment recovery) when support for reengaging in PA is most needed. Second, the program’s grounding in the SDT, a well-established behavioral framework, and incorporation of evidence-based strategies such as goal setting and self-monitoring with wearable activity trackers should assist participants with PA initiation and maintenance. Finally, the program’s delivery via telehealth addresses a significant access barrier for rural populations, which are often underrepresented in behavioral intervention research, while also facilitating the scalability and accessibility of OT.

Limitations include the use of a single-arm design, which constrains our ability to draw causal inferences about program effectiveness. The relatively small sample size (N=38) may also limit generalizability and statistical power to detect meaningful changes in PA and other outcomes. The reliance on self-report data for some measures introduces potential recall and social desirability biases. Finally, recruitment through local organizations and referrals may lead to a sample with higher baseline motivation (ie, selection bias), which could limit applicability to broader survivor populations.

### Conclusions

This study addresses a common and serious problem for individuals who have undergone breast conserving surgery or mastectomy: insufficient engagement in aerobic PA and MSE during the transition period from active treatment to everyday life. This health behavior theory–grounded program uses the expertise of occupational therapists to adapt, modify, and integrate these exercises into daily routines while delivering the program via telehealth (decreasing salient barriers to accessing supportive care) in a manner that is personally meaningful, thus increasing the likelihood of continued PA engagement post-program.

## Appendix

Multimedia Appendix 1 Exercise equipment catalog.

Multimedia Appendix 2 Peer review report from the American Cancer Society.

## Abbreviations

BC: breast cancer
BPNES: Basic Psychological Needs in Exercise Scale
HRQoL: health-related quality of life
IRB: institutional review board
MPI: multiple principal investigator
MSE: muscle-strengthening exercise
OT: occupational therapy
OUHS: The University of Oklahoma Health Sciences
PA: physical activity
PRO: patient-reported outcome
PROMIS: Patient-Reported Outcomes Measurement Information System
REDCap: Research Electronic Data Capture
SB: sedentary behavior
SDT: Self-Determination Theory

## Data Availability

Due to the pilot nature of this study and potential for participant identification, data from this study will not be shared with outside entities.

## References

[1] American Cancer Society (2022). Cancer treatment and survivorship facts & figures.

[2] Campbell K, Winters-Stone K, Wiskemann J, May A, Schwartz A, Courneya K, Zucker DS, Matthews CE, Ligibel JA, Gerber LH, Morris GS, Patel AV, Hue TF, Perna FM, Schmitz KH (2019). Exercise guidelines for cancer survivors: consensus statement from international multidisciplinary roundtable. Med Sci Sports Exerc.

[3] Braithwaite D, Satariano WA, Sternfeld B, Hiatt RA, Ganz PA, Kerlikowske K, Moore DH, Slattery ML, Tammemagi M, Castillo A, Melisko M, Esserman L, Weltzien EK, Caan BJ (2010). Long-term prognostic role of functional limitations among women with breast cancer. J Natl Cancer Inst.

[4] Zhu J, Wang F, Shi L, Cai H, Zheng Y, Zheng W, Bao P, Shu X (2020). Accelerated aging in breast cancer survivors and its association with mortality and cancer recurrence. Breast Cancer Res Treat.

[5] Joaquim A, Leão I, Antunes P, Capela A, Viamonte S, Alves AJ, Helguero LA, Macedo A (2022). Impact of physical exercise programs in breast cancer survivors on health-related quality of life, physical fitness, and body composition: evidence from systematic reviews and meta-analyses. Front Oncol.

[6] Phillips SM, McAuley E (2014). Physical activity and quality of life in breast cancer survivors: the role of self-efficacy and health status. Psychooncology.

[7] Smith SG, Chagpar AB (2010). Adherence to physical activity guidelines in breast cancer survivors. Am Surg.

[8] Ottenbacher A, Yu M, Moser R, Phillips S, Alfano C, Perna F (2015). Population estimates of meeting strength training and aerobic guidelines, by gender and cancer survivorship status: findings from the health information national trends survey (HINTS). J Phys Act Health.

[9] Forbes C, Blanchard C, Mummery WK, Courneya K (2015). Prevalence and correlates of strength exercise among breast, prostate, and colorectal cancer survivors. Oncol Nurs Forum.

[10] Schmitz KH, Campbell AM, Stuiver MM, Pinto BM, Schwartz AL, Morris GS, Ligibel JA, Cheville A, Galvão DA, Alfano CM, Patel AV, Hue T, Gerber LH, Sallis R, Gusani NJ, Stout NL, Chan L, Flowers F, Doyle C, Helmrich S, Bain W, Sokolof J, Winters-Stone KM, Campbell KL, Matthews CE (2019). Exercise is medicine in oncology: engaging clinicians to help patients move through cancer. CA Cancer J Clin.

[11] Ryan RM, Deci EL (2000). Self-determination theory and the facilitation of intrinsic motivation, social development, and well-being. Am Psychol.

[12] Deci EL, Ryan RM (2008). Self-determination theory: a macrotheory of human motivation, development, and health. Can Psychol.

[13] Ziviani J (2015). Occupational performance: a case for self-determination. Aust Occup Ther J.

[14] Dwyer LA, Hornsey MJ, Smith LGE, Oei TPS, Dingle GA (2011). Participant autonomy in cognitive behavioral group therapy: an integration of self-determination and cognitive behavioral theories. J Soc Clin Psychol.

[15] Hunter E, Gibson R, Arbesman M, D'Amico M (2017). Systematic review of occupational therapy and adult cancer rehabilitation: part 1. Impact of physical activity and symptom management interventions. Am J Occup Ther.

[16] Sheeran P, Wright C, Listrom O, Klein W, Rothman A (2023). Which intervention strategies promote the adoption and maintenance of physical activity? Evidence from behavioral trials with cancer survivors. Ann Behav Med.

[17] Hirschey R, Bryant AL, Macek C, Battaglini C, Santacroce S, Courneya KS, Walker JS, Avishai A, Sheeran P (2020). Predicting physical activity among cancer survivors: meta-analytic path modeling of longitudinal studies. Health Psychol.

[18] National Cancer Institute (2021). State cancer profiles: quick profiles—Oklahoma.

[19] Hegel MT, Lyons KD, Hull JG, Kaufman P, Urquhart L, Li Z, Ahles TA (2011). Feasibility study of a randomized controlled trial of a telephone-delivered problem-solving-occupational therapy intervention to reduce participation restrictions in rural breast cancer survivors undergoing chemotherapy. Psychooncology.

[20] Hwang N, Jung Y, Park J (2020). Information and communications technology-based telehealth approach for occupational therapy interventions for cancer survivors: a systematic review. Healthcare (Basel).

[21] Smith-Turchyn J, Gravesande J, Sabiston C (2020). Exercise interventions for survivors of cancer living in rural or remote settings: a scoping review. Rehabil Oncol.

[22] Czajkowski SM, Powell LH, Adler N, Naar-King S, Reynolds KD, Hunter CM, Laraia B, Olster DH, Perna FM, Peterson JC, Epel E, Boyington JE, Charlson ME (2015). From ideas to efficacy: the ORBIT model for developing behavioral treatments for chronic diseases. Health Psychol.

[23] Mazzoni A, Brooke HL, Berntsen S, Nordin K, Demmelmaier I (2021). Effect of self-regulatory behaviour change techniques and predictors of physical activity maintenance in cancer survivors: a 12-month follow-up of the Phys-Can RCT. BMC Cancer.

[24] Mazzoni A, Carlsson M, Berntsen S, Nordin K, Demmelmaier I (2019). "Finding my own motivation"—a mixed methods study of exercise and behaviour change support during oncological treatment. Int J Behav Med.

[25] Cheema BS, Kilbreath SL, Fahey PP, Delaney GP, Atlantis E (2014). Safety and efficacy of progressive resistance training in breast cancer: a systematic review and meta-analysis. Breast Cancer Res Treat.

[26] Henriksson A, Johansson B, Radu C, Berntsen S, Igelström H, Nordin K (2021). Is it safe to exercise during oncological treatment? A study of adverse events during endurance and resistance training—data from the Phys-Can study. Acta Oncol.

[27] Schmitz KH, Troxel AB, Cheville A, Grant LL, Bryan CJ, Gross CR, Lytle LA, Ahmed RL (2009). Physical Activity and Lymphedema (the PAL trial): assessing the safety of progressive strength training in breast cancer survivors. Contemp Clin Trials.

[28] Wilson P, Rogers W, Rodgers W, Wild T (2006). The psychological need satisfaction in exercise scale. J Sport Exerc Psychol.

[29] Hertzog MA (2008). Considerations in determining sample size for pilot studies. Res Nurs Health.

[30] Patel A, Friedenreich C, Moore S, Hayes S, Silver J, Campbell K, Winters-Stone K, Gerber LH, George SM, Fulton JE, Denlinger C, Morris GS, Hue T, Schmitz KH, Matthews CE (2019). American College of Sports Medicine roundtable report on physical activity, sedentary behavior, and cancer prevention and control. Med Sci Sports Exerc.

[31] Lambert JD, Greaves CJ, Farrand P, Cross R, Haase AM, Taylor AH (2017). Assessment of fidelity in individual level behaviour change interventions promoting physical activity among adults: a systematic review. BMC Public Health.

[32] Turner-Stokes L (2009). Goal attainment scaling (GAS) in rehabilitation: a practical guide. Clin Rehabil.

[33] Braun V, Clarke V (2006). Using thematic analysis in psychology. Qual Res Psychol.

[34] Leon AC, Davis LL, Kraemer HC (2011). The role and interpretation of pilot studies in clinical research. J Psychiatr Res.

[35] Thabane L, Ma J, Chu R, Cheng J, Ismaila A, Rios LP, Robson R, Thabane M, Giangregorio L, Goldsmith CH (2010). A tutorial on pilot studies: the what, why and how. BMC Med Res Methodol.

[36] Vlachopoulos SP (2008). The basic psychological needs in exercise scale: measurement invariance over gender. Struct Equation Model Multidisciplinary J.

[37] Vlachopoulos SP, Asci FH, Cid L, Ersoz G, González-Cutre D, Moreno-Murcia JA, Moutão J (2013). Cross-cultural invariance of the basic psychological needs in exercise scale and need satisfaction latent mean differences among Greek, Spanish, Portuguese and Turkish samples. Psychol Sport Exerc.

[38] Vlachopoulos SP, Michailidou S (2006). Development and initial validation of a measure of autonomy, competence, and relatedness in exercise: the basic psychological needs in exercise scale. Meas Phys Educ Exerc Sci.

[39] Vlachopoulos SP, Ntoumanis N, Smith AL (2010). The basic psychological needs in exercise scale: translation and evidence for cross‐cultural validity. Int J Sport Exerc Psychol.

[40] Kaminsky LA, Ozemek C (2012). A comparison of the actigraph GT1M and GT3X accelerometers under standardized and free-living conditions. Physiol Meas.

[41] Trost S, McIver K, Pate R (2005). Conducting accelerometer-based activity assessments in field-based research. Med Sci Sports Exerc.

[42] Troiano R, Berrigan D, Dodd K, Mâsse LC, Tilert T, McDowell M (2008). Physical activity in the United States measured by accelerometer. Med Sci Sports Exerc.

[43] Chevance G, Golaszewski NM, Tipton E, Hekler EB, Buman M, Welk GJ, Patrick K, Godino JG (2022). Accuracy and precision of energy expenditure, heart rate, and steps measured by combined-sensing Fitbits against reference measures: systematic review and meta-analysis. JMIR Mhealth Uhealth.

[44] Waddell A, Birkett S, Broom D, McGregor G, Harwood AE (2024). Validating the Fitbit Charge 4© wearable activity monitor for use in physical activity interventions. J Sci Med Sport.

[45] US Department of Health and Human Services (2018). Physical Activity Guidelines for Americans, 2nd edition.

[46] Papadopoulou C, Kotronoulas G, Schneider A, Miller M, McBride J, Polly Z, Bettles S, Whitehouse A, McCann L, Kearney N, Maguire R (2017). Patient-reported self-efficacy, anxiety, and health-related quality of life during chemotherapy: results from a longitudinal study. Oncol Nurs Forum.

[47] Cella D, Choi SW, Condon DM, Schalet B, Hays RD, Rothrock NE, Yount S, Cook KF, Gershon RC, Amtmann D, DeWalt DA, Pilkonis PA, Stone AA, Weinfurt K, Reeve BB (2019). PROMIS adult health profiles: efficient short-form measures of seven health domains. Value Health.

[48] Terwee CB, Peipert JD, Chapman R, Lai J, Terluin B, Cella D, Griffiths P, Mokkink LB (2021). Minimal important change (MIC): a conceptual clarification and systematic review of MIC estimates of PROMIS measures. Qual Life Res.

