# Supplementary material for: Digitally Mediated Occupational Therapy to Increase Physical Activity in Urban and Rural Breast Cancer Survivors: Protocol for a Single-Arm Feasibility Trial
Source: JMIR Res Protoc. 2025 Sep 26;14:e73554. doi: 10.2196/73554 (PMC12514410; doi:10.2196/73554)
Supplement: Multimedia Appendix 1 [file resprot_v14i1e73554_app1.pdf]

EXERCISE EQUIPMENT

- YOU HAVE $100 "STUDY BUCKS" TO SPEND ON EXERCISE EQUIPMENT.
- WORK WITH YOUR TEAM TO DECIDE WHAT IS THE BEST FIT FOR YOU!

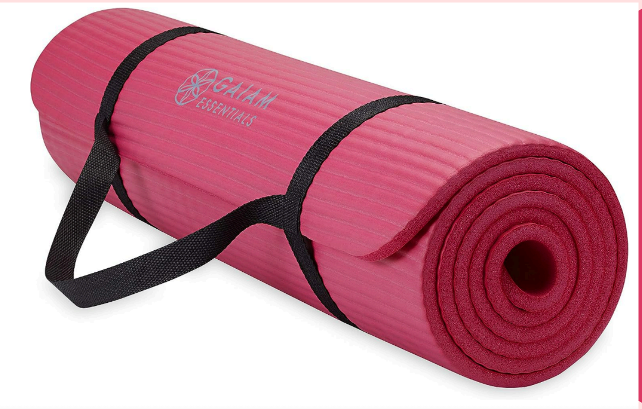

YOGA OR EXERCISE MAT: $25

Durable and grippy surface to stretch and exercise. Available in various colors.

STABILITY BALL: $25

Used for balance exercises and greater muscle activation during workouts.

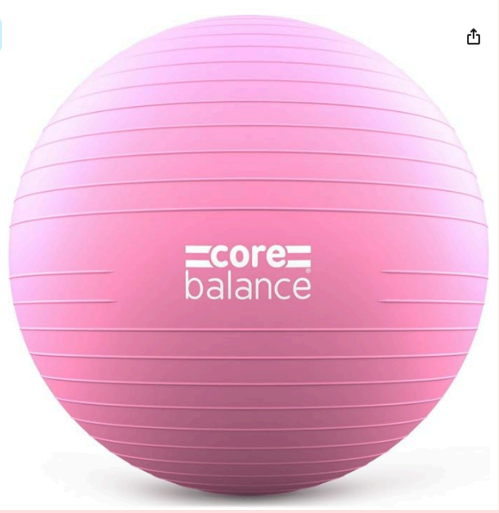

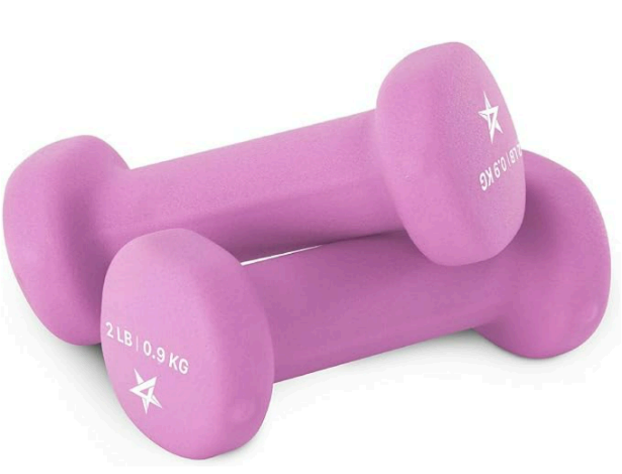

HAND WEIGHTS: $15 - $40*

Also known as dumbells or free weights, hand weights are useful for many different types of upper body exercise. Choose weights from 3 lbs. - 20 lbs.

RESISTANCE BANDS: $30

These stretchy bands provide a unique form of strength training that is different than traditional weightlifting.

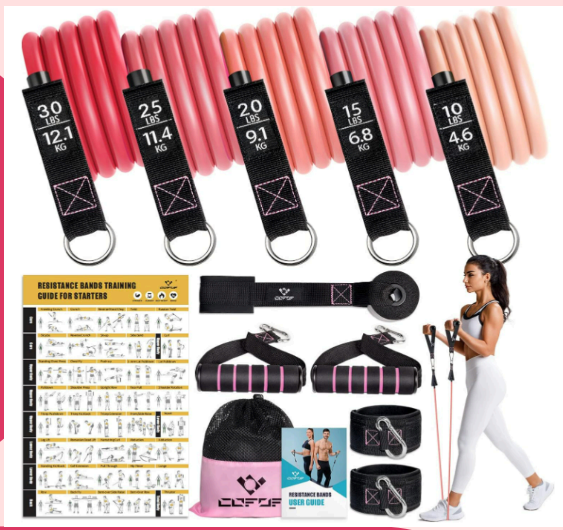

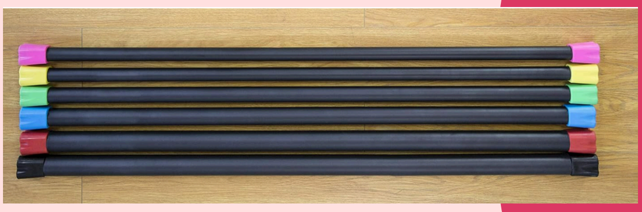

WEIGHTED RODS: $20-$40*

These versatile exercise tools are used for strength training, rehabilitation, and improving range of motion. Choose weights from 10 lbs. - 20 lbs.

Remember, exercise is essential for maintaining your physical and mental well-being, and it's important to prioritize taking care of yourself in your daily routine.

*price depends on weight selected

IRB APPROVED
IRB NUMBER: 16601
IRB APPROVAL DATE: 09/23/2024

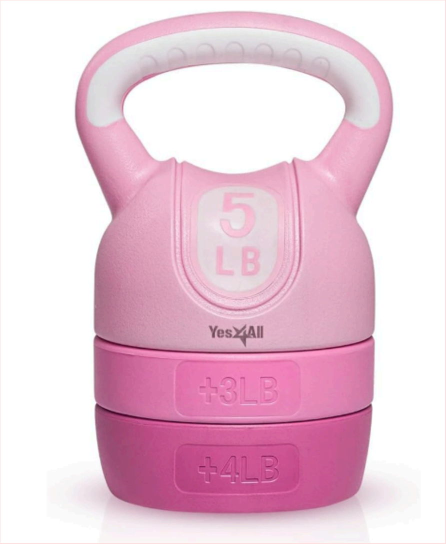

ADJUSTABLE KETTLEBELL: $35

Offers multiple weights and exercise options, including rows, swings, lifts, and presses. Comfortable and secure grip.

LARGE YOGA BOLSTER: $40

Supportive prop for yoga and stretching to provide comfort, enhance poses, and promote relaxation.

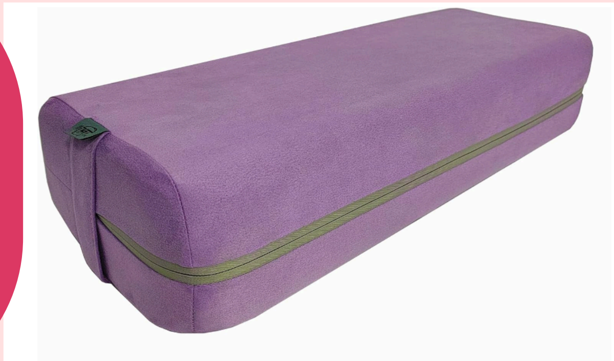

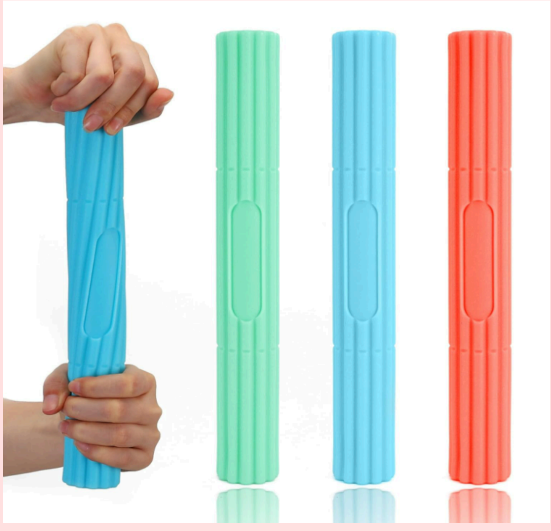

FLEX BARS: $35 PER SET

Rubber cylinder with a textured surface for twisting. Used to improve range of motion, strenghten muscles and tendons and improve grip strength.

GUASHA MASSAGE STONE: $15

Tool used for improving circulation, relieving muscle tension, reducing inflammation, and promoting relaxation.

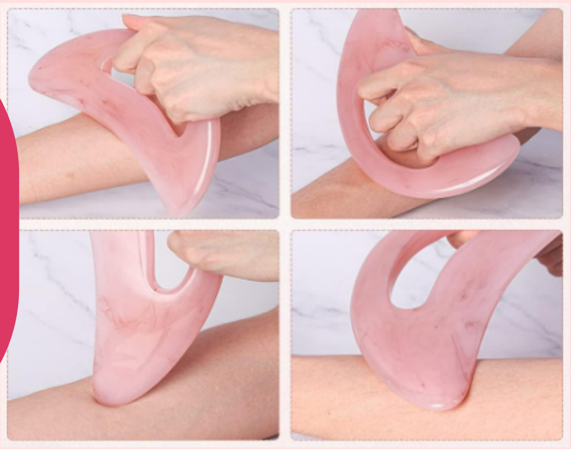

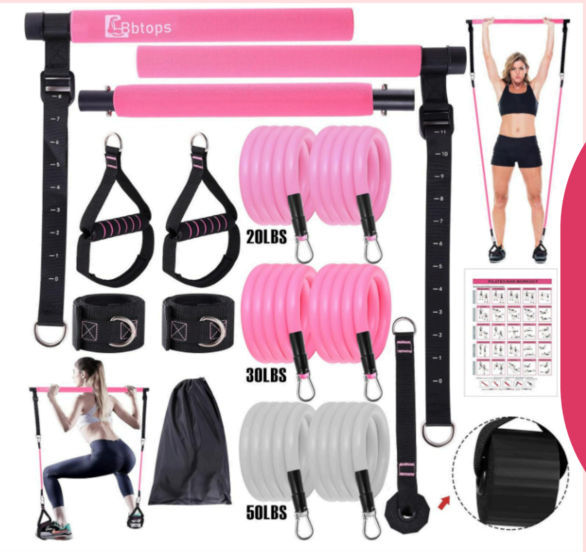

PILATES BAR KIT: $40

Consists of a bar and resistance bands. Promotes a full body workout by targeting several muscle groups, including the core, arms, legs, and back.

DRY BRUSH: $5

Dry brushing is a technique that is used to promote circulation and stimulation of the lymphatic system to improve lymphedema.

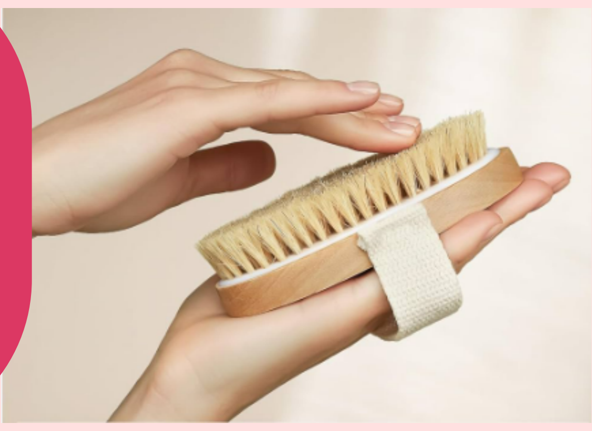

Remember, exercise is essential for maintaining your physical and mental well-being, and it's important to prioritize taking care of yourself in your daily routine.

IRB NUMBER: 16601
IRB APPROVAL DATE: 09/23/2024

LONG HANDLED DRY BRUSH: $15

Dry brushing is a technique that is used to promote circulation and stimulation of the lymphatic system to improve lymphedema. Long handle allows you to reach difficult areas.

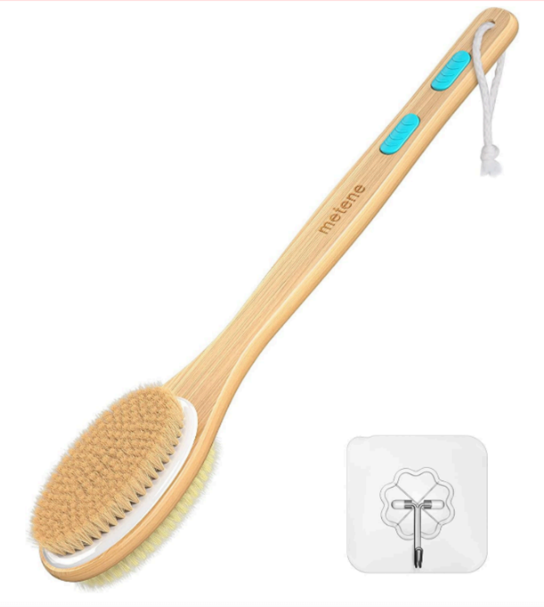

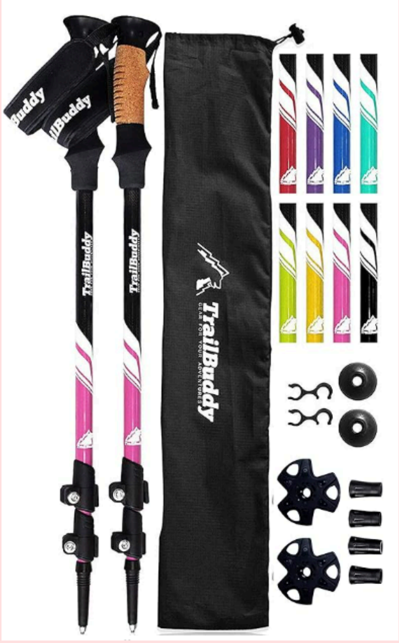

TREKKING POLES: $35

With adjustable height and comfortable grip, trekking poles provide support with balance during walking.

ARMBAND PHONE HOLDER: $10

Armband with space for phone, wired headphones, and a house key. Firm arm grip keeps your items handy during workouts.

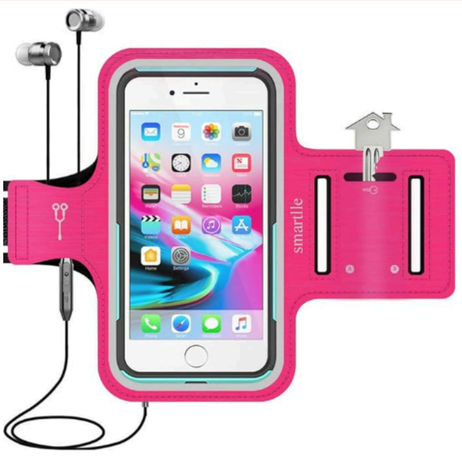

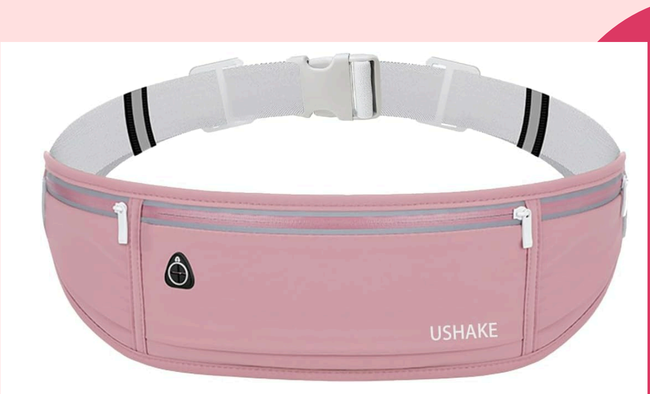

BELT PHONE HOLDER: $10

Wear this belt around your waist to store your phone, house key, and other small items during exercise.

YOGA HEADBAND: $5

Non-slip, wide, stretchy headband for yoga or exercise. Comfortable to wear, it won’t hurt your head after hours of use. Choose black or surprise Boho print.

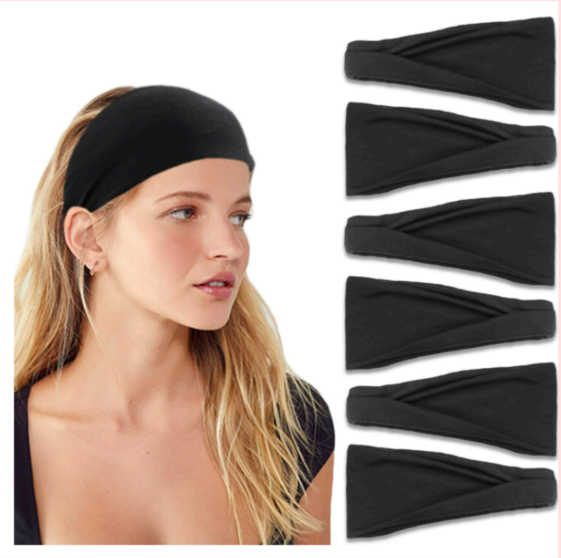

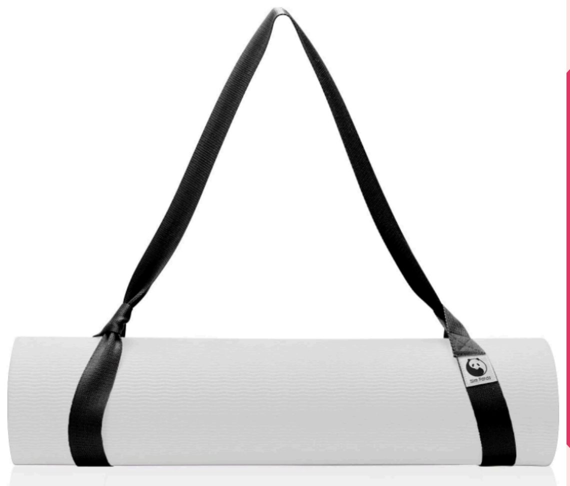

YOGA MAT STRAP: $5

The yoga mat strap will help you travel with your yoga mat. Fully adjustable to fit any size mat. Strap doubles as yoga prop to deepen stretches.

Remember, exercise is essential for maintaining your physical and mental well-being, and it's important to prioritize taking care of yourself in your daily routine.

IRB APPROVED
AAHRPP
IRB NUMBER: 16601
IRB APPROVAL DATE: 09/23/2024
