# Supplementary material for: Digitally Mediated Occupational Therapy to Increase Physical Activity in Urban and Rural Breast Cancer Survivors: Protocol for a Single-Arm Feasibility Trial
Source: JMIR Res Protoc. 2025 Sep 26;14:e73554. doi: 10.2196/73554 (PMC12514410; doi:10.2196/73554)
Supplement: Multimedia Appendix 2 [file resprot_v14i1e73554_app2.pdf]

PRINCIPAL INVESTIGATOR: Klinedinst, Tara

APPLICATION TITLE: A Digitally Mediated Occupational Therapy Program to Increase Physical Activity in Urban and Rural Breast Cancer Survivors Who Have Undergone Breast-Conserving Surgery or Mastectomy

SUMMARY:

This project seeks to assess a telehealth plan to deliver occupational therapy to post-surgical breast cancer patients in Oklahoma. This study is highly relevant to the SCC catchment area since approximately one third of breast cancer survivors live in rural areas which requires travel to access OT services and thus resents a barrier to adequate care. The PI has extensive experience implementing telehealth approaches in the older adult population, and seeks to transition this experience to breast cancer survivors. The research team is highly qualified, there is a high chance of successful completion of the project, and the project is likely to generate data that will support additional grant applications and high-quality publications. The only identified major weaknesses are the failure to articulate why an eight-week telehealth program is used and that the small sample size may not allow for analysis of some of the variables discussed in the proposal.

REVIEWER 1

1. **OVERALL IMPACT OF PROPOSED RESEARCH**

   This proposal focuses on breast cancer survivors who have a need for OT and physical activity after treatment. Up to 30% of Oklahoma breast cancer survivors live in rural areas and the time/distance to visit OT in clinic is one barrier to these people not receiving the kind of care that would benefit them. The PI has experience as an OT implementing telehealth programs to older adults to maintain their PA and will transition this type of work to breast cancer survivors in collaboration with experts in cancer and exercise physiology. Evidence is strong that PA can improve physical health and quality of life in cancer survivors and this project aims to make that possible for more people. The methods, aims, analysis, approaches are clearly described with milestones and objectives listed throughout the application. The results of the study could inform on potentially unique barriers faced by cancer survivors and better ways to motivate/educate them towards PA. Given that these are cancer patients, it could be beneficial to potentially re-evaluate the needs of patients after the baseline assessment that screens anxiety/depression and possibly address mental health needs as part of the telehealth program.

2. **SIGNIFICANCE**

   This project focuses on occupational therapy for breast cancer survivors in Oklahoma and aims to deliver care through an 8-week telehealth plan. The data are established that physical activity and OT can benefit patients during/after cancer treatment both physically and psychologically. However, not everyone has access to this option, nor do they realize it is effective. Delivery of this kind of treatment through telehealth is feasible and the goal is to determine what motivates people to alter their behavior with OT. The population of cancer survivors is growing and any increase in quality of life after cancer treatment would be a benefit. Motivation is hard to determine for some people and represents one barrier to adhering to OT or PA programs. If achieved, this study could provide some evidence that circumventing the barriers of time and distance to OT clinic can benefit rural Oklahoma breast cancer survivors (up to 30% of the population). It isn't clear if participant access to exercise tools or time for activity will be evaluated which may be one of the major barriers to adherence. Also, if depression turns out to be a major influence on feasibility in this population (as opposed to other groups, such as older adults) it might help to provide mental health care as part of the program.

3. **APPROACH**

   The research approach is described with Aim 1 to assess the perception of usefulness of the described OT telehealth program and Aim 2 to determine the feasibility and safety of the program. There is an exploratory aim to analyze certain variables pre to post program, with the hypothesis that changes will be favorable (improved PA, more usefulness of perception, etc). Plans for recruitment, inclusion/exclusion criteria are clear and potential attrition has been addressed and extra recruitment is planned in that case. Fit Bits will be provided, and the PI will have access to the data to better understand if participants are completing the project goals. Qualitative and quantitative assessments are described and planned carefully. Overall feasibility of the program will be assessed based on quantification of participants adherence and participation. This appears to be a project in the early stages with regards to breast cancer survivors but could provide strong preliminary data for future applications.

4. **INVESTIGATOR QUALIFICATIONS**

   The PI and team are qualified to carry out the proposed study. The PI is currently performing a similar study in older adults with the goal of helping them maintain PA remotely. The PI is a certified OT and has a bachelor's in psychology, and collaborators have expertise in breast cancer, exercise physiology, and implementation science, all in human studies. Together, they are experienced to help this unique population.

5. **CAREER DEVELOPMENT**

If successful, this project could shed light on how to better serve the needs of rural and urban Oklahoma cancer survivors who are not meeting PA recommendations or who have trouble transitioning back to PA after cancer surgery/treatment. This information could be used to create more in-depth assessments and then begin to implement ways to address the challenges faced in these unique populations as part of future proposals.

6. **CATCHMENT AREA RELEVANCE**

The application is focused on the distribution of rural and urban breast cancer survivors in Oklahoma, emphasizing the 30% rural participants who may not have adequate access to OT and PA training for cancer recovery. It will recruit directly from our catchment area and address the unique needs of the population.

7. **STATISTICS (Provide comments as necessary, but a trained biostatistician will also review this aspect of the application)**

Plans for data analysis are described. There are qualitative and quantitative assessments, and it is noted in the exploratory aim that the study is not powered to detect differences. Based on the type of trial this is, there is a paragraph describing how to handle missing data and how differences between categories will be measured and presented.

REVIEWER 2

1. **OVERALL IMPACT OF PROPOSED RESEARCH**

This proposed project seeks to provide digitally mediated occupational therapy to breast cancer survivors. A strength of the project is the focus on individuals within rural areas of Oklahoma that lack access to occupational health facilities. Promotion of physical activity to breast cancer survivors may provide improvements in quality of life and physical functioning. A moderate weakness of the project is whether the proposed eight-week period is sufficient to collect the anticipated data.

2. **SIGNIFICANCE**

- This proposed project focuses on breast cancer survivors and how physical activity can improve health-related quality of life.
- There is a need to develop digitally mediated occupational therapy, particularly for breast cancer survivors in rural areas who lack access to OT facilities.
- The proposed project has the potential to improve quality of life and physical functioning for breast cancer survivors.

3. **APPROACH**

- 38 participants (urban: rural ratio 77%:33%) will be recruited. Breast cancer survivors within OKC and OK.
- Inclusion and exclusion criteria are clearly stated.
- Unclear why eight weeks of telehealth-delivered occupational therapy (OT) are sufficient for this study. What is the rationale for the eight-week period?

4. **INVESTIGATOR QUALIFICATIONS**

- PI Klinedinst is a research-trained occupational therapist and assistant professor at OUHSC with a good track record in securing research funding and publications.
- Overall, a great team of collaborators and mentors with relevant expertise.

5. **CAREER DEVELOPMENT**

- The proposed project has the potential to inform the scale-up and implementation of the current program using the NIH Stage Model to guide our decision-making.
- There are plans to submit an R01-level proposal. However, no details are provided about which specific program might be targeted.

6. **CATCHMENT AREA RELEVANCE**

33% of Oklahoma is classified as rural. There is a need to engage rural populations in clinical studies, which aligns with the SCC priorities.

7. **STATISTICS**

REVIEWER 3

1. **OVERALL IMPACT OF PROPOSED RESEARCH**

The PI proposes to study the acceptability, feasibility, safety, and potential effect of a novel, telehealth-delivered OT program grounded in Self-Determination Theory (SDT) for aerobic PA and MSE among BC survivors in both rural and urban settings. Proposed research is seeking to promote physical activity primarily in rural breast cancer survivors through a digitally delivered, health behavior theory-informed occupational therapy (OT) program. As numerous barriers (e.g., time burden, distance to the OT clinic, economic issues, etc.,) can preclude rural and some urban breast cancer survivors from participating in OT post-treatment, the observations from this study could have far-reaching implications for breast cancer patient's quality of life, well-being, and better survival.

Some minor concerns with recruitment of BC patients post-surgical (both breast conserving and mastectomy) randomization with just 15 patients from rural areas.

2. **SIGNIFICANCE**

Strength:

- PI will study the feasibility of telehealth-based health promotion in BC survivors undergone a Mastectomy. The focus will be on rural patients with limited access to OT due to time/distance.
- This proposal is improving the breast cancer patient's quality of life through the aerobic PA and MSE, which is highly significant.

Weakness: None

3. **APPROACH**

Strength:

- Study design is well described. Inclusion/exclusion criteria, % of patients, timeline etc.
- Main hypothesis is built based on inaccessibility of patients to OT due to remote dwelling. 1/3rd of patients will be recruited from rural areas.
- Definition of rurality at county level will be followed while enrolling.
- Will use technologies like video calling, PA tracking using fit bit.
- The timeline of the project is provided, and the goals are achievable (if patient accrual is successful).
- Proposed to publish 2 articles.
- Dr. Henson, an oncologist provided support letter and confidence of accruing 5 patients/month.

Weaknesses:

- No discussed the alternative plan enrollment could not be achieved in the given time frame.
- Given the small sample size, assessing several variables discussed (sociodemographic sec F.3.4) may not be achievable.
- Will training be provided to the patients on using video calling/Fitbit usage/setup.
- How or what is the novelty of the 8-week OT program is not discussed.

4. **INVESTIGATOR QUALIFICATIONS**

Strength:

- PI is licensed OT and Asst Prof. Other two PD/PI have complementary expertise needed for this project. Well qualified for this project. Supported by Mentor and Radiation oncologist.
- PI-Robertson's experience/NCI funded research award F31 was on similar work in Breast cancer survivors, may considered as additional strength.

Weakness:

- Plan to hire another Occupational therapist (overlapping expertise with PI). Time needed for Hiring OT, but not discussed how additional funds will be committed in hiring this position.

5. **CAREER DEVELOPMENT**

- If successful, is completion of this project likely to enhance the career development of the PI? Will the project provide the investigator with preliminary data for additional grant applications? Are future directions/plans clearly articulated?

Strength:

- RO1 is in preparation with similar kind of work. Already have a pilot grant from PHF to generate preliminary data. Data from this proposed study will provide additional strength to the application.

Weakness: None

6. **CATCHMENT AREA RELEVANCE**

Strength:

- Study design aims to cover all racial/ethnic groups representative of OK population.
- Plan to enroll extra patients to substitute for patients who withdraw/non-compliant.
- The proposal deals with qualify of life improvement in breast cancer survivors belonging to underrepresented group/ living in rural/remote areas with no access to OT. Hence it is relevant to the SCC catchment area.

Weakness:

- Minor but project mainly addresses single gender (female patients) since breast cancer is very rare in male.

7. **STATISTICS**

Proposed to use 30 breast cancer patients (15 rural and 15 urban) seems appropriate statistical analysis. Need a biostatistician to review this aspect.

BIOSTATISTICS REVIEW

Aim 1 will consist of semi structured interviews with qualitative analysis of these interviews using standard methodology. A usability score will also be calculated from a scale and the means, and 95% confidence interval will be calculated. For aim 2 feasibility and safety will be assessed by calculating the proportion of participants that meet certain goals/criteria. There are also some exploratory aims where they will conduct descriptive statistics and 95% confidence intervals to help inform future studies. All analyses are appropriate.
